# Do It Yourself (DIY) Radiolucent Drill Guide Using a 10 ml Syringe for Freehand Distal Interlocking in Intra-medullary Nailing: A Surgical Technique

**DOI:** 10.7759/cureus.49216

**Published:** 2023-11-22

**Authors:** Chezhiyan Shanmugam, James O Smith

**Affiliations:** 1 Trauma and Orthopaedics, Dorset County Hospital, Dorchester, GBR

**Keywords:** diy, surgical technique, intra-medullary nailing, freehand distal interlocking, radiolucent drill guide, do it yourself

## Abstract

Distal interlocking during intramedullary femoral, tibial, and humeral nailing is frequently challenging. In the traditional image intensifier (II) 'bull's eye' technique, the implant's interlocking screw hole can be obscured by the radio-opaque chuck, necessitating multi-planar checks by tilting the drill bit before drilling. This manoeuvre can adversely alter the drill trajectory, compromise fixation, or damage the implant. We introduce a surgical technique that uses a 10 ml syringe to overcome this difficulty.

## Introduction

The study explores a novel surgical technique designed to address the challenges associated with distal interlocking during intramedullary nailing procedures in the femur, tibia, and humerus. The traditional 'bull's eye' technique using image intensifiers often poses difficulties due to the obscuration of the implant's interlocking screw hole by the radio-opaque chuck, leading to the need for multiple checks and potential complications during drilling. In response to these challenges, we introduce a cost-effective and innovative method using a disposable 10 ml syringe to facilitate precise distal interlocking. This technique involves drilling through the syringe and using it as a radiolucent guide for accurate positioning, eliminating the need for repeated image intensifier checks. This approach not only reduces the radiation exposure risk but also accelerates the surgical procedure, making it a valuable addition to the existing repertoire of distal interlocking techniques.

We have previously published an alternative technique using a Dynamic Hip Screw guide wire to make a pilot hole prior to using the proprietary drill. The technique presented here enhances this approach and may obviate the need for a pilot hole due to the increased accuracy of drilling. The technical tip emphasizes significant reductions in X-ray exposures and recommends the integration of this approach for its cost-effectiveness and consistent reproducibility. This adoption is envisioned to enhance both patient safety and surgical efficiency.

## Technical report

The distal interlocking hole within the nail is identified using image intensifier in the standard fashion, and a small incision is made down to the bone (Video 1).

**Video 1:** Do it yourself radiolucent drill guide using a 10 ml syringe Do it yourself (DIY) radiolucent drill guide using a 10 ml syringe for freehand distal interlocking in intra-medullary nailing.

**Figure 1:**
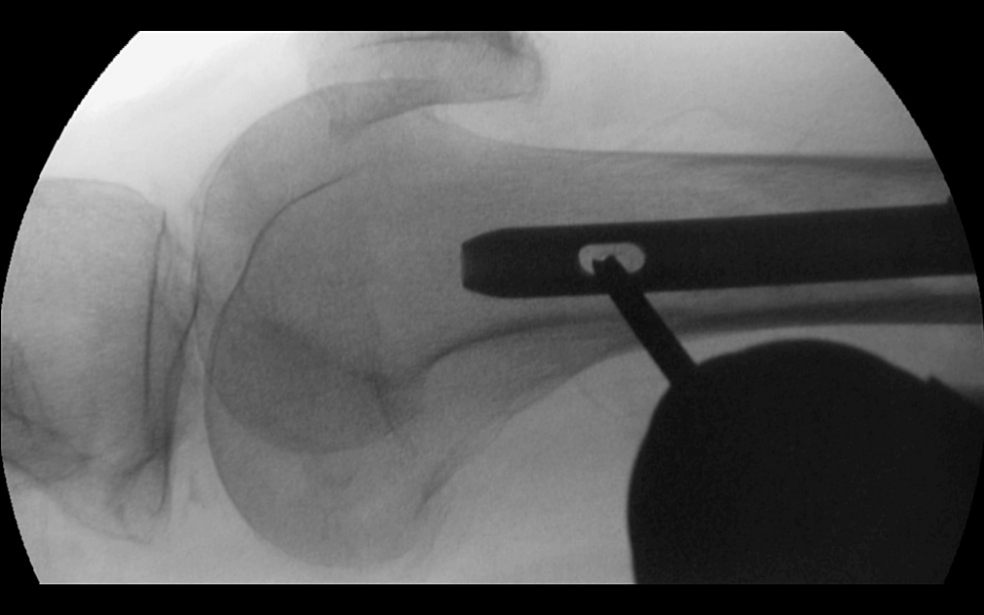
Conventional chuck The conventional chuck obscures the trajectory of the interlocking hole drill. This can be mitigated by our DIY (Do It Yourself) radiolucent drill guide technique.

The traditional 'bull's eye' technique using image intensifiers often poses difficulties due to the obscuration of the implant's interlocking screw hole by the radio-opaque chuck, leading to the need for multiple checks and potential complications during drilling (Figure 1).

**Figure 2:**
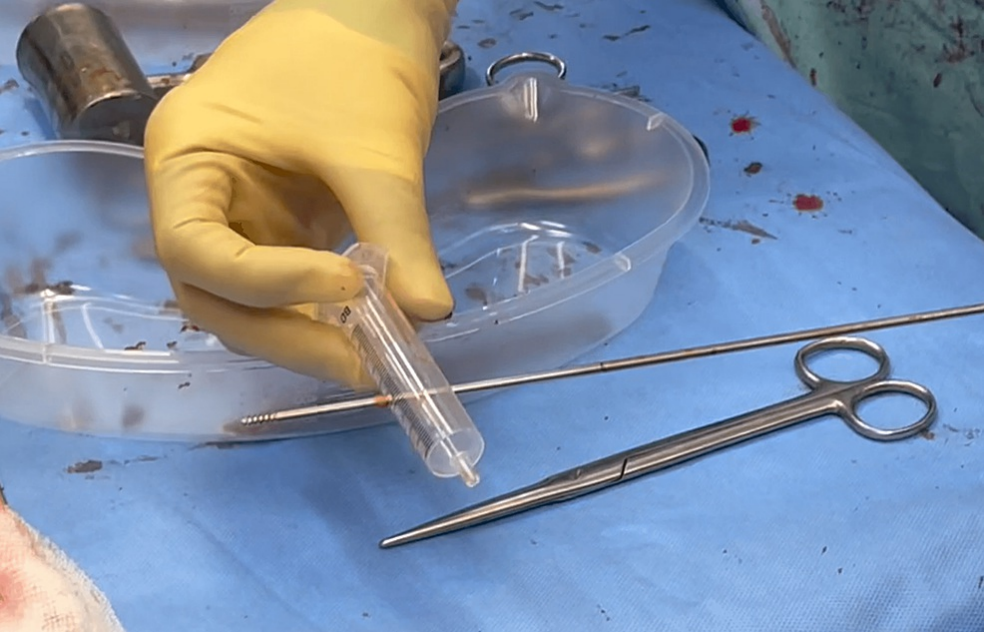
Preparation of radiolucent kit A disposable 10 ml syringe is prepared with the drill bit.

To overcome this difficulty, a disposable 10 ml syringe is prepared as the drill bit support: the internal plunger is withdrawn to its outer end to serve as a handle. Then, the intended interlocking drill bit is used to drill transversely through the barrel of the syringe at its distal third (Figure 2).

A steristrip is applied over the junction of the drill bit and the quick coupling is incised using a blade at its junction. This will serve as a marker for alignment and for re-engagement once the freehand technique establishes the correct trajectory.

**Figure 3:**
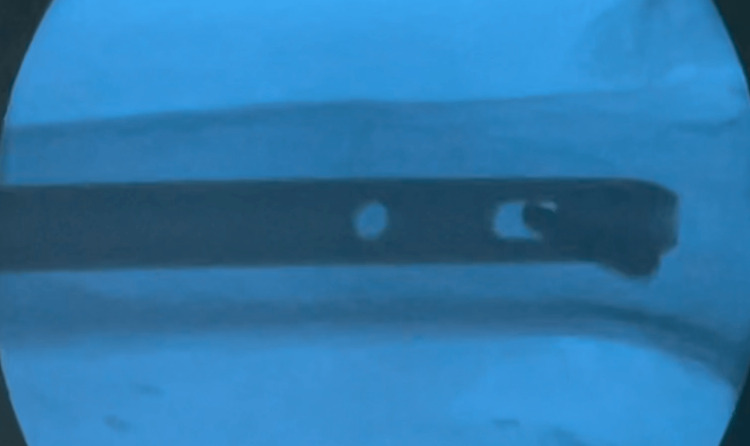
Radiolucent kit in action. Image intensifier film shows that once the drill chuck is uncoupled, the syringe with an embedded drill bit is a radiolucent kit.

Once the drill chuck is uncoupled, the syringe with an embedded drill bit serves as a radiolucent kit, with the withdrawn plunger acting as a handle distant from the intensifier field. The drill bit can be placed in the correct orientation within the bony window and checked with the image intensifier. This helps to visualize only the drill bit, as the syringe is radiolucent, and at this point, no chuck is attached (Figure 3).

**Figure 4:**
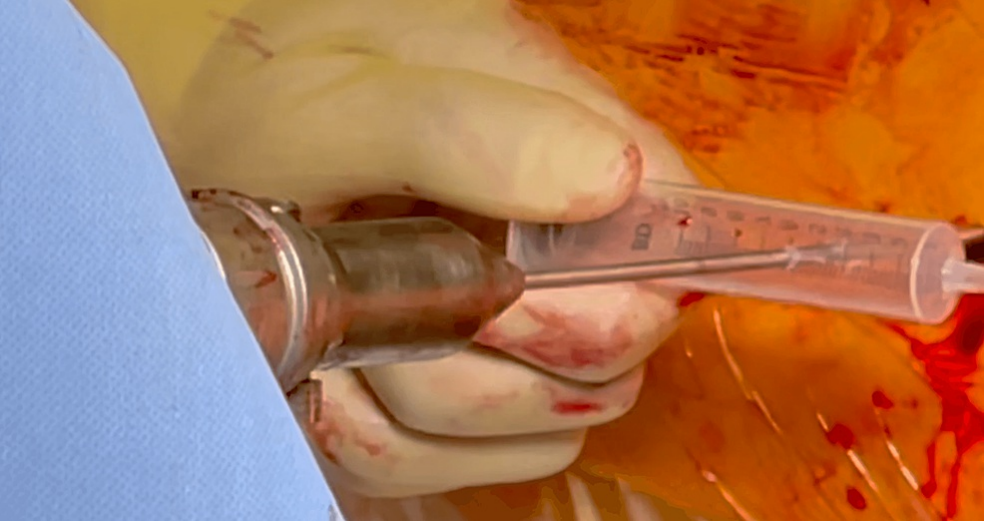
Syringe serves as a handle. The internal plunger is withdrawn to its outer end can serve as a handle retaining the trajectory.

This gives good control of the drill bit, helping to localize the 'bull's eye' of the interlocking hole and placement of the drill in its correct version without the need for a multiplanar check Thereafter, the drill may be quickly coupled by the surgical assistant, who matches the steristrip markings while the surgeon holds the drill bit in its correct version. This reduces the error margin associated with a conventional technique and removes the cost of more cumbersome interlocking kits offered by implant manufacturers. The operator may choose to ignore the step of application of steristrip if the surgeon remembers the drill bit slot position in the drill chuck (Figure 4).

## Discussion

Various techniques have been published to simplify the distal interlocking process. Ritesh Kumar Soni et al. [1] published radiation-free insertion of the distal interlocking screw in tibial and femur nailing - a simple technique using a nail-on-nail technique. Michael Perrone et al. have reported proximal interlocking in a retrograde nail using a 3 ml cut syringe as a radiolucent guide. This involves a minimal access surgical approach in the proximal thigh and carries the risk of damage to nearby neurovascular structures [2]. Sithombo Maqungo et al. compared 'Distal Interlocking Screw Placement in the Femur: Free-Hand Versus Electromagnetic Assisted Technique (Sureshot)', which required an electromagnetic kit to perform, which may add to further cost available from Smith and Nephew [3, 4].

Similarly, distal targeting systems are available from Stryker, which also involves additional costs [5]. Shanmugam and Smith published a pilot hole technique using a dynamic hip screw (DHS) guide wire prior to using the proprietary drill. The use of this method might eliminate the necessity for employing a pilot hole with a DHS** **guide wire [6].

Cost-effective analysis and safety

The bulk purchase cost of a 10 ml syringe is £0.19 (approx. $0.20) in the open market. This compares favourably with the cost of the proprietary distal targeting jig - the market price is £3487 from Stryker, and the Smith and Nephew Trigen Sureshot loan kit costs approximately £660 plus the cost of disposables.

## Conclusions

We noted a significant reduction in the number of image intensifier checks, thereby reducing the radiation risk and speeding up the procedure. We noted a reduction of X-ray exposures by an average of five shots when compared with using conventional radiopaque drills. We recommend this technique as it is cost-effective and consistently reproducible, decreasing the operative time, which directly reduces infection risk, yet achieving the same or superior precise distal interlocking using the widely performed freehand technique.
